# 5-years outcomes following arthroscopic anterior cruciate ligament reconstruction comparing quadruple hamstring and peroneus longus tendon autografts: a randomized control trial

**DOI:** 10.1007/s00402-024-05639-1

**Published:** 2024-12-23

**Authors:** Umer Butt, Filip Vuletic, M Ali Ahmed Shaikh, Amanullah, Ghufran ur Rehman, Imran Ali Shah, Anders Stålman, Zainab Aqeel Khan

**Affiliations:** 1 Department of Trauma and Orthopaedic, AO Hospital, Karachi, Pakistan; 2 Sulis Hospital Bath, Bath, UK; 3 Department of Orthopaedics, Kantonsspital Baselland, Basel, Switzerland; 4 Department of Molecular Medicine and Surgery, Stockholm Sports Trauma Research Center, Karolinska Institutet, Valhallavägen 91, Stockholm, Sweden; 5 University Hospital “Sveti Duh”, Zagreb, Croatia; 6 Faculty of Kinesiology, University of Zagreb, Zagreb, Croatia; 7 Department of Orthopaedic Surgery, Hayatabad Medical Complex, Peshawar, Pakistan; 8 Dalian Medical University, Dalian, China; 9 Capio Artro Clinic Sophiahemmet Private Hospital, Stockholm, Sweden

**Keywords:** ACL reconstruction, Peroneus longus, Autograft, Hamstring autograft, Sports injury, Sports surgery

## Abstract

**Aims:**

This study presents clinical outcomes, functional results, and return to sports after anterior cruciate ligament (ACL) reconstruction using quadruple hamstring tendon autograft or peroneus longus tendon autograft in a randomized controlled trial.

**Patients and methods:**

Between February 2018 and July 2019, patients who underwent ACL reconstruction were randomly assigned to two groups: hamstring and peroneus longus. Patient related outcome measurements and pain intensity were evaluated using IKDC, Lysholm, and visual analog scores at 3 and 6 months, 1, 2, and 5 years after the surgery. At the 5 year follow-up, anterior stability was tested using the 3D printable Knee Arthrometer. In addition, in the peroneus longus group, ankle functional assessment was performed using the American foot and ankle score. Additionally, data on the return to sports/activities was collected for both groups at the last follow-up.

**Results:**

Sixty patients, with 30 in the hamstring group and 30 in the peroneus group, were included in the study. Patients were predominately male and with low activity demands. After five years of follow-up, there was no significant difference in functional assessment scores (IKDC and Lysholm) between the two groups (P *n.s*). The median graft diameter was 7.9 ± 0.4 mm in the hamstring tendon group and 8.9 ± 0.2 mm in the PL group (P < 0.001). The improvement in Arthrometer testing measurements (AMT) for the operated knees in the hamstring and peroneus longus groups were similar. In the peroneus longus group, the mean postoperative foot and ankle score was 98.6 ± 3.9 (range = 85–100).

**Conclusion:**

Using Peroneus longus tendon autograft for arthroscopic ACL reconstruction is a feasible alternative as studied in this cohort of predominately male patients with low activity demands. The graft diameter in this study was sufficient, and the results regarding laxity and patient related outcome measurements were similar to those achieved with hamstring tendon autografts.

**Level of evidence:**

Level I

**Trial registration:**

ChiCTR2000036989

## Introduction

Arthroscopic anterior cruciate ligament (ACL) reconstruction is the gold standard treatment for instability following ACL injuries [1]. Bone-patellar tendon-bone (BPTB), quadruple hamstring tendons, and quadriceps tendon autografts are some of the most commonly used graft choices for ACL reconstruction. BPTB graft is often suggested for high-demand patients who wish to return to high-impact sports earlier [2, 3]. However, the risk of anterior knee pain is a well-known complication of BPTB graft [3]. Pain-free kneeling is a significant concern in some sports and in some religious groups who kneel when praying [4]. Hamstring grafts are popular due to easy harvesting and similar outcomes to BPTB autografts with less donor site morbidity [5]. On the other hand, the decline in hamstring muscle power after tendon harvest is a potential problem for athletes such as sprint runners [6]. Persistent muscular weakness and laxity is common postoperatively [7–9].

Among the various graft options available, utilizing the peroneus longus tendon for ACL reconstruction has gained attention in recent years [10]. The peroneus longus (PL) tendon, located in the lateral compartment of the lower leg. Unlike other popular autografts harvested from the knee, the PL tendon autograft offers some advantages in reducing residual donor site morbidity. Bypassing the knee joint avoids potential problems such as quadriceps and hamstring weakness [11, 12]. The harvesting technique for the PL tendon has been described in detail and it can be easily extracted because it works synergistically with the peroneus brevis muscle to fold over the ankle joint [12]. Current literature, presenting good clinical outcomes, supports using PL tendon as a graft for ACL reconstruction [10, 13–15]. However, the literature does not provide explicit answers regarding the direct comparison of outcomes and failure rates between PL tendon and other graft options and longer-term follow-up is lacking [13].

Therefore, we aimed to conduct a randomized control trial to evaluate and compare the 5 year clinical and functional outcomes of PL tendon and the commonly used hamstring tendon autografts for ACL reconstruction. We hypothesized that the PL tendon is a feasible option for ACL reconstruction with outcome comparable to hamstring tendon grafts.

## Patients and methods

This prospective randomized control trial was conducted in the Orthopaedic Institute AO Hospital sports injury unit from December 2018 to July 2023. Consolidated Standards of Reporting Trials (CONSORT) guidelines were used, and all the procedures followed the Helsinki Declaration. The ethical approval was obtained from the hospital’s ethical review board (UMDC/Ethics/2016/15/06/391), and the trial was registered in the Chinese clinical trial registry with the registration number ChiCTR2000036989. The informed consent was taken from all patients before enrolment in the study. All patients were followed up for at least five years postoperatively.

The study included patients who were 18 years or older and had a confirmed symptomatic primary ACL rupture on magnetic resonance imaging (MRI) scan, as well as a positive Lachman’s and anterior drawer test. Patients with a multi-ligament knee injury, chondral damage needing surgical treatment, meniscal injury requiring repair, hyperlaxity defined as a Beighton score > 4 and ACL reconstruction on the contralateral knee were excluded. Additionally, we excluded patients with any laxity, fracture, or previous surgery at the ankle joint. During the study inclusion period, a total of 78 patients underwent arthroscopic primary ACL reconstructions by a single sports surgeon. Sixty-four patients met the inclusion criteria for this study. The patients were randomly assigned to two groups: reconstruction with quadruple hamstring tendon autograft (n = 33) or a PL tendon autograft (n = 31). The randomization was achieved using a computer-generated algorithm. Patients were assigned to groups using non-probability consecutive sampling. The choice of graft (quadruple Hamstring or PL tendon) was not disclosed to the operating surgeon or the patient before the surgery. It was only revealed in the operating theatre just before the operation. After the surgery, patients became aware of the graft choice due to the visible incision.

## Surgical technique

Arthroscopy was performed in a supine position under regional or spinal anaesthesia. Before starting the procedure, an examination under anaesthesia (EUA) was carried out, and a pivot shift test was performed to assess knee instability. The tourniquet was then applied to the thigh and inflated. Standard anterolateral and anteromedial portals were used, and diagnostic arthroscopy was completed before reconstruction, followed by graft harvest for the ipsilateral hamstring tendons or PL tendon.

For PL tendon graft harvesting, a 2 cm incision was made 3 cm above the tip of the fibula. The tendon sheath was identified through a longitudinal incision over the tendons, gently exposing both the peroneus longus and brevis (PB). The distal end of PL was sutured to the brevis using a vertical mattress suture with three mattresses sutured on each side. The tenodesis of PL to PB was followed by placement of Lahey’s forceps under the PL whip stitch initiated using a 2-0 Ethicon suture (Johnson & Johnson Medical Belgium). With the help of a closed tendon stripper, the proximal end of the tendon was released from its proximal musculotendinous attachment. The detailed technique for PL tendon graft harvest has recently been published [18].The Semitendinosus and Gracilis were harvested through a small incision on the proximal medial tibial aspect. After harvesting and graft preparation, a double PL and quadruple Semitendinosus and Gracilis construct were fixed to the femoral aspect with the adjustable ACL Tight rope (Arthrex Naples, FL, USA) technique and a bio-composite screw (Arthrex Naples, FL, USA) for graft fixation at the tibial aspect.

### Post-operative rehabilitation

Following the surgery, a hinged knee brace was applied to the patient for two weeks. They were advised to use crutches for ten days and allowed to bear weight as tolerated with a full range of knee motion. Starting the first day after the surgery, the patient was instructed to perform isometric quadriceps training, straight leg raises, active flexion, and extension. By the fourth week, the patient was allowed to perform strengthening exercises for quads and hamstrings with light weights.

## Outcome measures

Outcomes were measured at three months, six months, 1, 2, and 5 years postoperatively in the outpatient department by an independent orthopaedic surgeon (other than the operating surgeon) using the International Knee Documentation Committee (IKDC) and Lysholm knee score. Reliable and valid tools for assessing knee function after knee injury or surgery [19, 20]. Questions about return to previous level of activities were asked. The severity of pain was evaluated using a visual analogue (VAS) score [21]. The knee stability after reconstruction was assessed by Lachman’s anterior drawer and pivot shift test in both groups intra-operatively and at three months follow-up after surgery. The diameter of the graft was measured intra-operatively and compared between the two groups. Anterior stability in 20° of flexion was tested by the manual 3D printable Knee Arthrometer (Orthopedic Skills Laboratory of the Health Sciences, Department of the Federal University of Paraná in conjunction with the Engineering Department of the Federal Technological University of Paraná) at five years follow-up. The laxity of the operated knee was measured in both groups and compared with the non-operated knee for comparison [16, 17, 22].

Thigh circumference was measured at 7 cm above the patella in centimetres (cm). The American Foot and Ankle Score (AFAS) was used to assess the ankle function after PL graft harvesting [23].

## Sample size calculation and statistical analysis

The sample size was calculated by using minimal clinically significant difference (MCID) for IKDC (11.5 points) [19]. The standard deviation of IKDC postoperatively was extracted from the literature at a confidence interval (CI) of 95% [14]. The total estimated sample size suggested 15 participants in each group. However, we included 30 participants in each group to account for the possible loss of follow-up.

Descriptive statistics were used to measure each variable’s mean and standard deviations. The normality of the data was checked using the Shapiro–Wilk test for all measurements and parametric or non-parametric statistical tests were chosen accordingly. The graft diameter between the two groups was compared using an independent T-test. The non-parametric Kruskal–Wallis test was used to compare postoperative functional scores and pain intensity after every follow-up. The non-parametric Mann–Whitney U test was used to compare functional outcome scores (IKDC and Tegner) and pain intensity between groups after five years of surgery. The Arthrometer testing measurements (AMT) were compared within groups (non-operated and operated knees) and between groups using the Mann–Whitney U test. The independent T-test was used to compare thigh circumferences between non-operated and operated knees. The level of significance was set at a P value < 0.05.

## Results

**Fig. 1:**
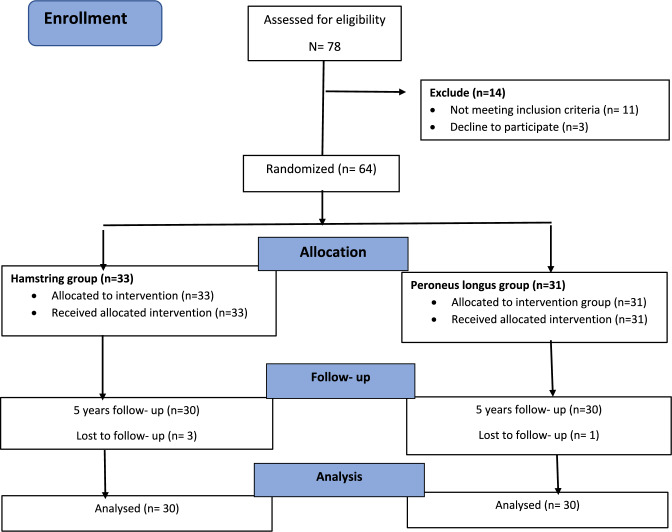
Flow chart of the study showing participants randomization and enrolment

Sixty-four patients met the inclusion criteria, but four were lost to follow-up, with three in the hamstring group and one in the PL group. Of the remaining 60 patients, 30 were in the hamstring group, and 30 were in the PL group. The mean age of participants was similar in the hamstring and PL groups, with 29.2 ± 5.0 years in the hamstring group and 27.7 ± 4.1 years in the PL group. All participants, except one female in the hamstring group, were male. All patients completed the last follow-up after five years The details of participants’ recruitment and inclusion are presented in the study flow chart (Fig. 1), and the baseline details of the study groups are reported in Table 1. The median graft diameter was 7.9 ± 0.4 mm in the hamstring tendon group and 8.9 ± 0.2 mm in the PL group. There was a statistically significant difference found between the two groups in graft diameters (P < 0.001). At three-month follow-up, all patients were examined for knee stability. No difference between the groups in Lachman’s anterior drawer and pivot shift test were found. The mean AMT for non-operated and operated knees in the hamstring group were 5.7 ± 1.3 and 5.8 ± 1.8, respectively with side-to-side mean difference of 0.14. Similarly, the PL group’s mean AMT was 5.3 ± 0. for non-operated and 5.0 ± 1.13 for operated knee, respectively, with side to side mean difference of 0.33. There were no statistically significant side-to-side differences found between groups.

**Table 1:** Demographics of study population

Variables	Hamstring (n = 30) N (%)	Peroneus longus (n = 30) N (%)
Age ± SD	29.17 ± 5.03 years	27.73 ± 4.14 years
Gender		
Male	29 (96.6)	30 (100)
Female	1 (3.3)	0
Side		
Right	17 (56.6)	12 (40)
Left	13 (43.3)	18 (60)
Mechanism of injury		
RTA	8 (26.6)	9 (30)
Sports injury	15 (50)	10 (33.3)
Fall	7 (23.3)	11 (36.6)
Menisectomy	6 (20)	10 (33)
Minor chondral damage (I–II ICRS) (%)	10 (33)	13 (43)

*RTA* road traffic accident, *N*/*A* not applicable, *ICRS* international cartilage repair society

**Fig. 2:**
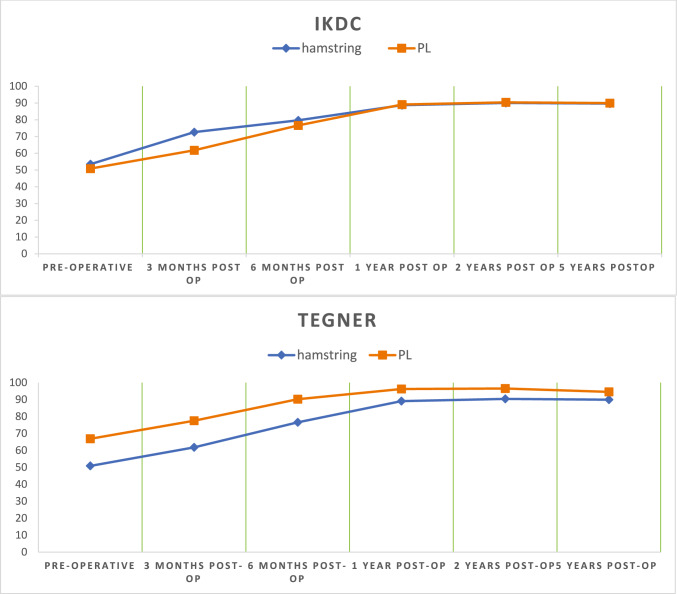
Comparison of functional outcomes (IKDC and Lysholm) between hamstring and PL group at different follow-up (pre-operative, 3,6 months, 1,2- and 5-years post-operative)

The functional outcomes (IKDC and Lysholm score) improved at follow-up in both groups compared with the preoperative scores, as presented in Fig. 2. There was a significant improvement between the preoperative and 5 year postoperative scores in both study groups (P < 0.001). However, there was no significant difference between the two groups for either IKDC and Lysholm at 5-year follow-up, as shown in Table 2. The VAS for pain decreased at each follow-up visit in both groups. The mean values for VAS after 5- years were similar; 0.50 ± 0.57 and 0.50 ± 0.62 in the hamstring and PL groups, respectively.

**Table 2:** Functional outcomes and pain intensity of hamstring and peroneus longus at 5 years follow-up

Scores	Groups	Pre-operative	5 years post- operative	Score change	P value
IKDC	Hamstring	53.4 ± 12.8	89.7 ± 5.7	36.3	< 0.001
	PL	50.8 ± 12.9	89.9 ± 9.8	39.1	< 0.001
	P value	0.5 8(n.s)	0.22 (n. s.)		
Tegner	Hamstring	62.1 ± 16.9	95.1 ± 5.2	33	< 0.001
	PL	66.8 ± 16.9	94.5 ± 6.4	27.7	< 0.001
	P value	0.10	0.86 (n. s.)		

*PL* peroneus longus, *n. s.* not significant

The mean thigh circumference for non-operated and operated knees was 37.3 ± 3.4 and 36.5 ± 3.2 cm, respectively in the hamstring group. Similarly, in the PL group the mean thigh circumference for non-operated and operated knees was 38.2 ± 2.8 and 38.2 ± 2.6 cm respectively. No statistically significant difference in thigh circumference was found in any of the groups.

In the hamstring group, 8 (27%) patients were involved in sporting activities before ACL injury. Among them, 6 (75%) patients returned to their pre-injury level within 12.33 ± 2.06 months after reconstruction. In the PL group, 7 (23%) patients were involved in sporting activities before ACL injury. All patients returned to their pre-operative level within an average of 11.7 months after reconstruction. All details regarding return to sports is reported and presented in Table 3.

**Table 3:** Return back to sports after ACL reconstruction

Participation in sports	Hamstring (n = 8) 30%	PL (n = 7) 23%
Sports played		
Cricket	4 (50)	3 (43)
Football	4 (50)	3 (43)
Badminton	–	1 (14)
Level of sports		
Recreational	8 (100)	6 (86)
National	None	1 (14)
Return to sports after surgery		
Yes	6 (75)	7 (100)
No	2 (25)	None
Time from surgery to return to sports (in months)	12.33 ± 2.06	11.7 ± 1.49
Return to sports at same pre-operative level		
Yes	6 (75)	7 (100)
No	2 (25)	None

The mean 5 year AFAS score in the PL group was 98.8 ± 4.04 (range = 80–100). Clinical examination of the ankle noted no difference in range of motion or instability between legs.

## Discussion

The most important finding of this study were that the PL tendon is a suitable alternative autograft for ACL reconstruction. The mean IKDC and Lysholm scores showed excellent clinical outcomes comparable to the more often used hamstring autograft, with no significant difference between groups at five-year follow-ups. Laxity postoperatively was also similar between the groups. Low donor site morbidity was found in the ankle joint.

The choice of graft for ACL reconstruction depends on the surgeon’s preference. However, it has been proposed that choice of graft should be individualized considering donor site morbidity, patient lifestyle and choice of activities [24]. The choice of autograft impact postoperative outcomes and need for post-operative rehabilitation. In their study, Wiradiputra et al. [25, 26] suggested that the PL tendon autograft for ACL reconstruction should be considered a first choice in ACL reconstruction due to low donor site morbidity and no biomechanical impact on the knee joint after harvesting the tendon. Rudy et al. found no significant difference in tensile strength between hamstring and PL tendon autografts, as concluded from biomechanical analysis [25]. Recent literature suggests that autograft diameter plays a crucial role in graft failure and re-rupture rate. Some studies implicate that grafts with a diameter less than 8 mm might be associated with increased risk of failure [27, 28]. The mean diameter of the PL tendon autograft is typically greater than 8 mm (ranging from 8–9 mm), which is larger than the hamstring tendon [29, 31]. In this study, the median diameter was 7.9 ± 0.8 mm in the hamstring tendon group, while it was 8.9 ± 0.7 mm for the PL tendon autograft. These data support the finding of a sufficient graft size with the use of PL. Further we found no re-rupture or graft failure cases during the 5 year follow-up period in either group but only few of our patients participated in sports.

Previous studies on the PL as an autograft in ACL reconstruction and a recent systematic review and meta-analysis by He et al. that analysed 23 studies on the PL tendon for ACL reconstruction reported that patients with PL tendon autograft showed equal mean scores of IKDC and Lysholm compared to hamstring and there was no difference in laxity between the groups [6, 13–15, 29, 30, 34]. In the present study, similar findings were found with a longer 5-year follow up, postoperative patient related outcome measurements and knee laxity was comparable.

Donor site morbidity should be discussed when the decision of graft choice is taken. Complications such as injury to the infrapatellar branch of the saphenous nerve and thigh hypotrophy are commonly reported after harvesting hamstring graft and the hamstrings are considered agonists of the ACL. These complications can result in hypoesthesia at the donor site and reduced hamstring strength, which can significantly impact postoperative rehabilitation and possibly the patient’s quality of life. Harvesting the PL could possibly affect ankle joint stability and strength [32]. Using PL tendon autograft for harvesting may result in associated donor site morbidities, such as reduced peak torque eversion and inversion and decreased ankle joint function and stability. Bi et al. therefore proposed using only the anterior half of the PL tendon and did not remove the complete tendon to avoid functional impairment at the ankle joint [13]. However, we were not able to find any difference in thigh circumference between the legs and groups indicating no hamstring hypotrophy and the AFAS score for perceived ankle function showed excellent results in this longer 5 year follow-up.

The finding of excellent ankle function after PL harvesting is supported by Rhatomy et al. [14, 33], who reported that harvesting the PL tendon does not affect ankle eversion and plantar flexion strength when compared to the contralateral healthy site.

It is important to note that our study has some limitations. The results presented are based on a single-centre trial with only one operating surgeon. Due to our study’s demographics, our findings cannot be generalized to the broader population, as no female patients were included. This was due to the social dynamics of a conservative society, where fewer females participate in sports and undergo reconstructive surgery after ACL injuries. The study showed similar patient related outcome measurements and also in the PL group excellent more specific ankle outcome related measurements, but no strength testing or other functional tests were performed. This study could not evaluate the risk of graft failure and re-rupture rates. Our patients were mostly low demand and only a few were active in sports before and after surgery. Therefore, we assumed this as a reason for the finding of no graft failure or re-rupture and the study cannot be generalized to a more active population.

To establish the use of PL tendon as a routine autograft for ACL reconstruction, further studies could focus on long-term outcomes, graft failure or re-rupture rates in more active populations. Impact on the ankle joint could be better assessed in functional tests and biomechanical analysis.

## Conclusion

In conclusion peroneus longus tendon autografts provided a sufficient graft diameter in ACL reconstruction. Similar laxity, and patient related outcomes were found in comparison to hamstring tendon autografts with low donor site morbidity in this study with predominantly male patients and low demand activity level. The peroneus longus tendon autograft can therefore be considered an option in ACL reconstruction with similar results as hamstring tendon autografts at a 5 year follow-up.

## Data Availability

The data supporting the findings of this study are available from the corresponding author upon reasonable request.

## Declarations

### Clinical trial registration

The trial was registered in the Chinese clinical trial registry with the registration number ChiCTR2000036989.
